# A case report: new-onset nummular headache after stenting of the middle cerebral artery

**DOI:** 10.1186/s12883-024-03552-z

**Published:** 2024-02-19

**Authors:** Yuanyuan Liu, Xuehong Chu, Xiao Dong, Erlan Yu, Xiaole Jia, Chuanjie Wu, Xunming Ji

**Affiliations:** 1 Department of Neurology, Xuanwu Hospital, Capital Medical University, Beijing, 100053 China; 2 Xuanwu Hospital, China-America Institute of Neuroscience and Beijing Institute of Geriatrics, Capital Medical University, Beijing, 100053 China; 3 Beijing Institute for Brain Disorders, Capital Medical University, Beijing, 100069 China; 4 The Fourth Clinical College, Capital Medical University, Beijing, 100000 China

**Keywords:** Nummular headache, Intracranial stent implantation, Secondary headache

## Abstract

**Background:**

Nummular headache (NH) is categorized as a primary headache in the International Classification of Headache Disorders, Third edition (ICHD-3) diagnostic criteria, but there are secondary etiologies as well. We present a case of secondary NH that associated with vascular lesion.

**Case presentation:**

We report on a 40-year-old man with a medical history of symptomatic intracranial arterial stenosis who developed a headache after percutaneous transluminal angioplasty and stenting because of Intracranial atherosclerotic stenosis(ICAS). This new-onset headache was a pinprick headache confined to the parietal part of the head and 5 cm in size. This headache most closely resembled the phenotype of a NH. And other causes of secondary headache were excluded. Thus, the diagnosis of NH was highly speculated. This patient represents a rare headache phenomenon after intracranial arterial stent placement.

**Conclusion:**

This is the first report of NH after stent placement treatment in a patient with ICAS.

## Background

Nummular headache (NH) (ICHD-3 code, 4.8) is a rare headache that is characterized by persistent or intermittent headache. And the pain is confined to a oval or elliptical-shaped area of 1–6 cm on the scalp. While NH is considered a primary headache disorder, secondary etiologies have been described. The lesions of the secondary NH can occur in the scalp, bone, trauma and blood vessels. A few cases of NH have been associated with vascular lesions, but these have been associated with fusiform areas of superficial vessels near the area of pain [1]. 

Pertinent to our case is the previous report by Dr. Ighodaro in neurology. They described a 31-year-old woman who developed new onset persistent severe NH immediately after stenting for an intracerebral aneurysm [2]. The phenomenon of stenting causing headache in patients with intracranial atherosclerotic stenosis(ICAS) is relatively common, but leading to typical NH is rare. So this case is the first report of NH after stenting in ICAS patients.

## Case presentation

With a medical history of hypertension, respiratory sleep apnea syndrome, and hyperlipidemia, a 40-year-old male presented to the outpatient neurology clinic for continuous headache on the parietal of the brain for 4 years. Because of the left middle cerebral artery(MCA) severe stenosis, he was treated with percutaneous transluminal angioplasty and stenting. After the operation, the patient immediately developed a headache. There was no history of headache prior to this operation. Written informed consent was obtained from this patient.

**Fig. 1:**
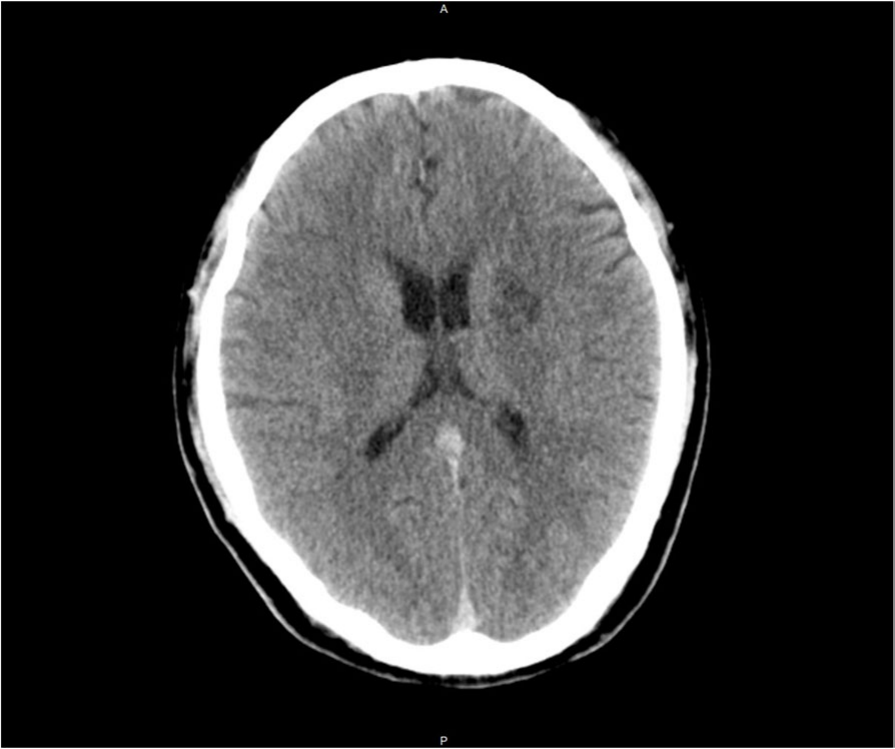
This is the patient’s CT imaging

**Fig. 2:**
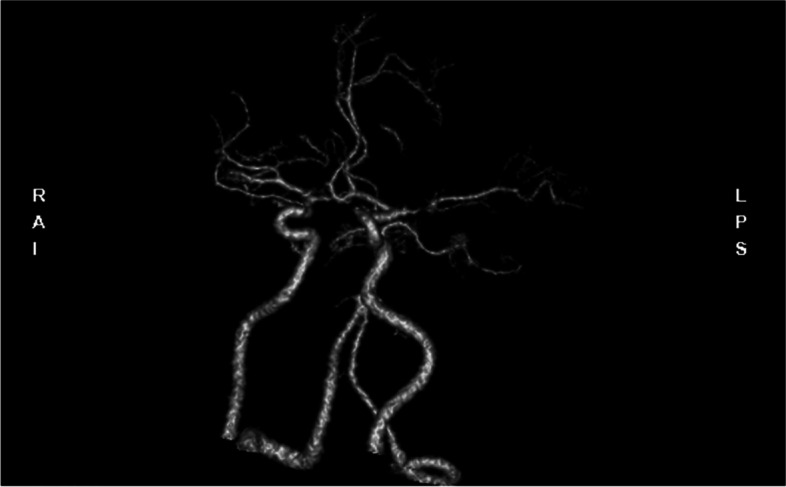
CTA imaging of the patient. It shows that severe stenosis of the MI segment of the middle cerebral artery

**Fig. 3:**
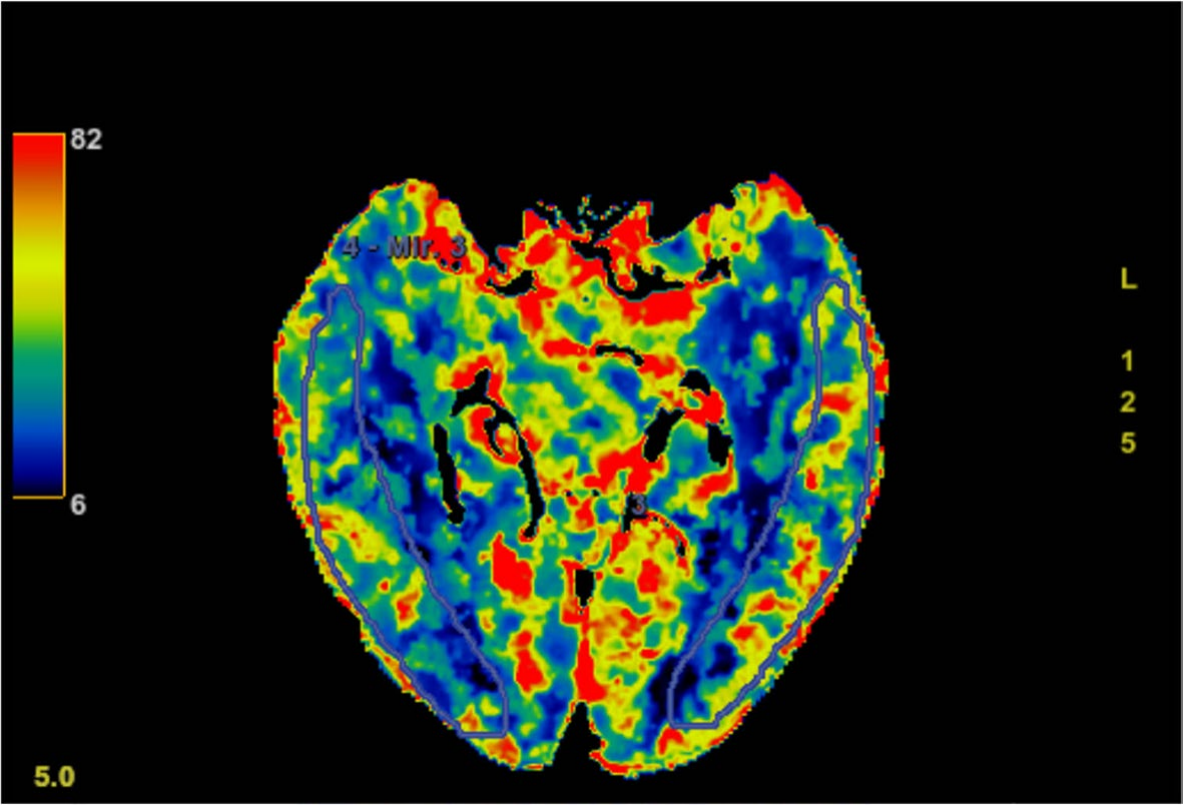
CTP imaging of the patient

**Fig. 4:**
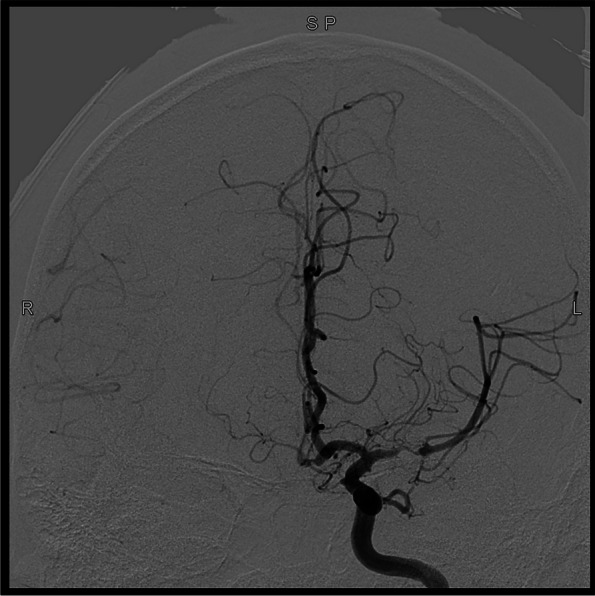
pre-operative angiograph imaging of the patient

**Fig. 5:**
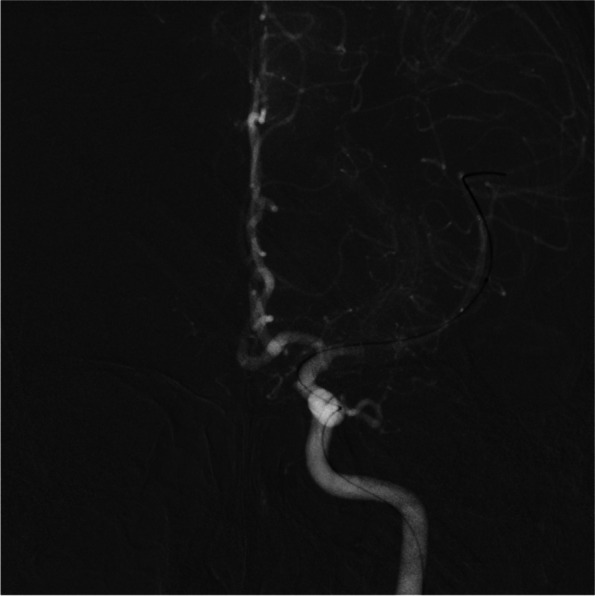
intraoperative angiograph imaging of the patient. It can be seen that the microguidewire passes through the M1 section

**Fig. 6:**
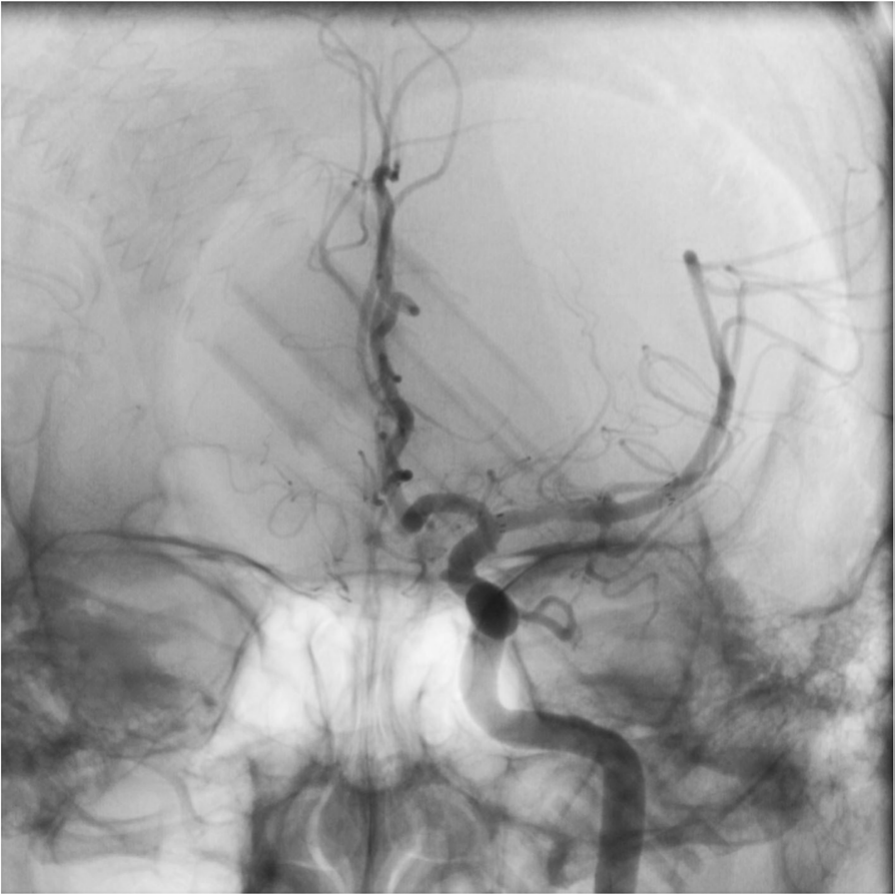
post-operative angiograph imaging of the patient. Blood flow can clearly be seen restored to the previously stenosed M1 segment

More than 4 years ago, without any cause, the patient developed weakness of the right side of the limbs in a quiet state. And this symptom lasted for 20 days. An MRI examination of the brain with and without contrast, a subsequent MR angiogram head without contrast and a DSA examination (Figs. 1, 2, 3, 4, 5 and 6) revealed acute cerebral infarction in the left basal ganglia and corona radiata, stenosis of the left MCA M1 segment, and mild atherosclerosis of the carotid arteries. So the patient was treated with aspirin and clopidogrel. Based on medical history and examination, the patient was considered symptomatic ICAS. So the local doctor recommended stent implantation treatment for him.

**Fig. 7:**
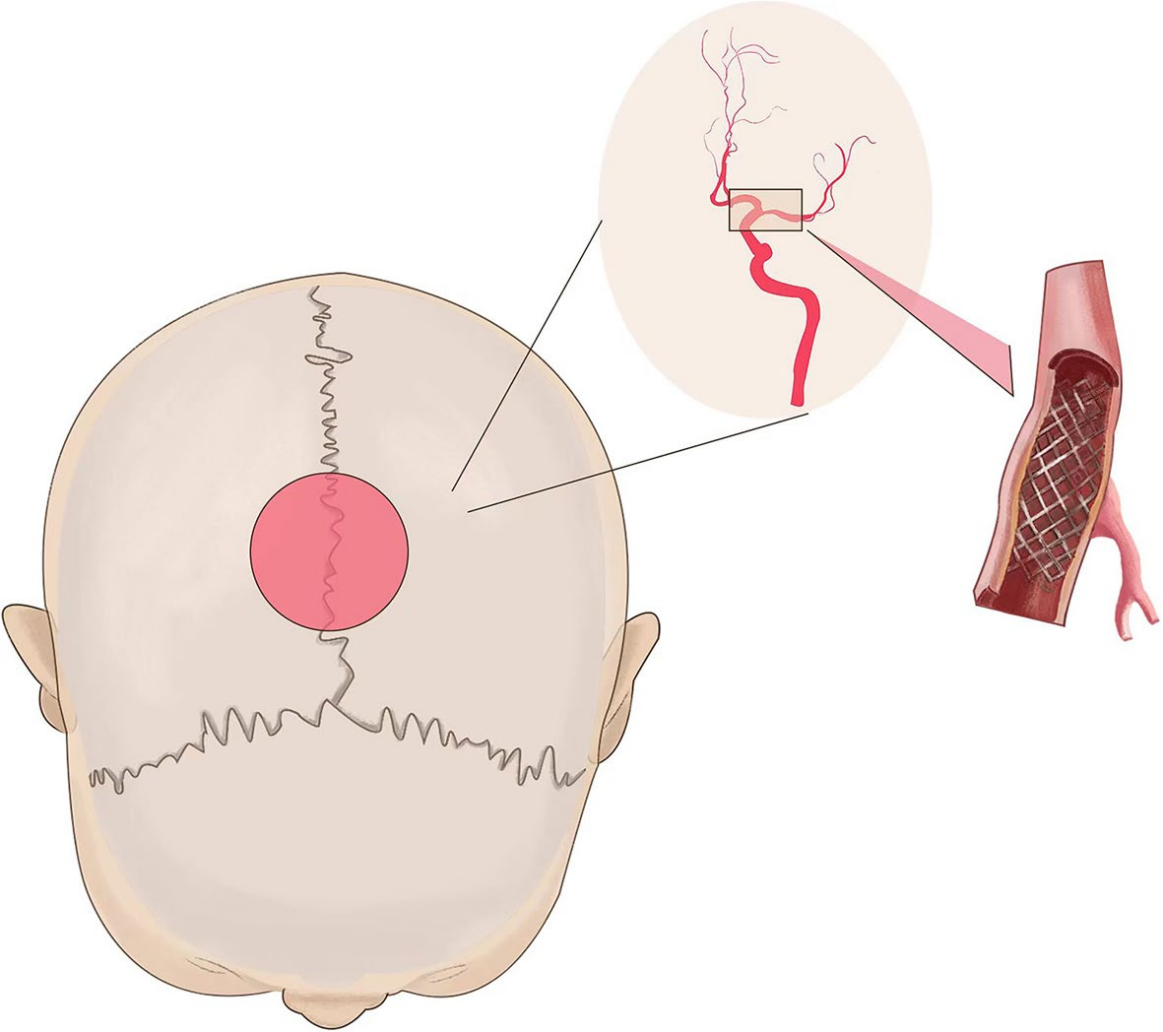
Pinpoint stabbing pain 5 cm in diameter in the region of the head due to stent implantation in the middle cerebral artery. The area of headache was shaped like a coin

Twenty-one days after the onset of the disease, under general anesthesia, the patient underwent stenting of the M1 segment of the left middle cerebral artery and percutaneous transluminal angioplasty. The left middle cerebral artery was first dilated with a 2.0 × 15 mm balloon and then implanted with a stent (4 × 20 mm, Neuroform EZ stent system, Stryker Corporation, USA). Immediately after awakening from general anesthesia, the patient described a persistent sharp pain localized to a circular, approximately 5 cm diameter in the parietal part of the brain. And this headache was not accompanied by nausea or vomiting, phonophobia, bulbar conjunctival congestion, or a runny nose (Fig. 7). The Visual Analogue Scale (VAS) score was five. There was no rash in the region and no skin/hair change. The pain was present all day without associated allodynia, frequent paresthesia. He would sometimes feel subjective warmth in the skin of the scalp in that region. In the clinic, his neurologic examination was unremarkable. By his description, this headache most closely resembled the phenotype of a NH, according to the ICHD-3.

The patient was seen in our clinic 4 years after the onset of focal headache. For abortive headache therapies, he had previously tried ibuprofen 2.4 g on 15 days per month. And this therapy was lasting for 2 months. The headache improved when taking the medication and persisted when not taking the medication. The patient was not taking preventative headache therapies. After regular treatment, the patient felt that the symptoms were not well relieved, so he gave up regular treatment and only took medication when he had severe pain.

During the clinic visit, he was diagnosed with nummular headache and started on a trial of gabapentin 300 mg per day. During a follow-up visit, he noted that the 300 mg of gabapentin daily was ineffective. And he did not experience any adverse effects from the medication during follow-up. For this reason, we recommend that patients increase the dose of gabapentin to 600 mg daily. And we advised the patient to re-visit the clinic if there is no significant improvement after the dosing.

## Discussion and conclusions

Within three months of the patient’s initial headache, the physician considered it to be a headache resulting from endovascular therapy(ICHD-3 code, 6.5.3) or a headache resulting from cerebral ischemia(ICHD-3 code, 6.1.1.1). And a recent study was conducted to prospectively evaluate the clinical characteristics of headache after thrombectomy. They found nearly one-third of the patients in the study experienced de novo headache after thrombectomy [3]. However, the patient did not improve after 3 days, so headache due to endovascular therapy was excluded. Moreover, the patient’s MRI examination of the brain did not reveal any new infarct foci, so headache due to cerebral ischemia was also excluded according to the diagnostic criteria of the ICHD-3 [4]. The patient did not have a prior history of migraine headaches(ICHD-3 code, 1), nor did he take regular analgesic for more than 3 months, so it was not a medication overuse headache either [4]. Apart from that, the patient had no history of trauma(ICHD-3 code, 5.2). And the detailed physical examination, especially the neurologic, cranial and five-sensory examinations, was unremarkable.

After examination found cerebral infarction, the patient was taking aspirin combined with clopidogrel. After stent implantation the patient has been taking aspirin 100 mg/day for secondary prevention of ischemic stroke. Although some studies have suggested that headaches can occur with overdose of aspirin (ICHD-3 code,8.2.3.2), the patient did not experience an overdose of the drug, so headaches from drugs used to treat cerebral infarction were ruled out. Excluding the secondary causes of headache considered above, we tentatively consider the patient’s headache to be a primary headache.

The patient suffered from persistent headache confined to a small circular area of the head, 5 cm in diameter. These features were consistent with a diagnosis of NH. NH is considered a rare disorder, and its true prevalence and incidence is uncertain. In a hospital-based study, the incidence of NH was 6.4/100,000 per year. After the first case was reported in 2002, and since then, more than 300 cases have been described [5]. Initially, NH is categorized as a primary headache in the ICHD-3. As in many other primary headache disorders, secondary cases may occur. The cases that have been reported so far are mainly associated with lesions of the scalp, bone, trauma, and blood vessels [5]. Regarding vascular lesions causing NH, Dr. Ighodaro previously reported in neurology. They found a 31-year-old woman who developed new-onset persistent intense NH immediately after stenting for an intracerebral aneurysm [2]. So we considered that our patient’s headache is similar with this case. The phenomenon of headache caused by stent placement in ICAS patients is relatively common, but there is no precedent for causing typical NH. So this case is the first report of NH after ICAS patients stenting. From these two cases, it is even more suggestive that intracranial arterial stenting may also be a possible etiology of NH [2]. 

This headache is due to the effects of local endovascular manipulation on the vasculature, possibly due to inflammation of the intracranial vessel wall caused by endothelial injury, which activates sensory afferents [6]. And related studies about coronary arteries after stent implantation have found that the stent mechanically disrupts the plaque, which leads to the release of a number of inflammatory factors at the target lesion. The inflammatory factors stimulate the production of acute phase proteins in the liver, and then the inflammatory factors and the acute phase proteins activate signaling pathways in the endothelium at the non-target lesion. The above mechanisms lead to persistent vascular inflammation [7]. So it is hypothesized that there may have been exsited chronic inflammation after stent implantation so that the patient presented with a persistent headache.

Currently, the anti-CGRP receptor monoclonal antibody Erenumab has been widely used as a prophylactic drug for migraine. This is because CGRP is an important peptide in the pathophysiology of orofacial pain. The patient is currently being treated with gabapentin 600 mg per day and if the treatment remains ineffective after increasing the dosage. We may consider treating the patient with Erenumab, an anti-calcitonin gene-related peptide (CGRP) receptor monoclonal antibody [8].

Endovascular treatment of cerebral infarction using stents is increasingly used. Headache after stenting are very common. And this treatment is sometimes associated with a number of serious complications in addition to headache. Therefore, additional evaluation is necessary for any patient with cerebral infarction who develops de novo headache after edovascular treatments. If the evaluation shows no structural abnormalities and the new postprocedural headache remains unexplained, secondary nummular headache due to post-stenting should be considered.

To the best of the author’s knowledge, this is the first report of NH after stent placement treatment in a patient with ICAS. Secondary nummular headache due to post-stenting should be considered.

## Data Availability

Data is provided within the manuscript.

## Abbreviations

NH: Nummular headache
ICHD-3: The international classification of headache disorders, third edition
ICAS: Intracranial atherosclerotic stenosis
MCA: Middle cerebral artery
VAS: Visual Analogue Scale
